# Observed discordance between outcomes reported by acromegaly patients and their treating endocrinology medical provider

**DOI:** 10.1007/s11102-019-01013-2

**Published:** 2019-12-05

**Authors:** Eliza B. Geer, Jill Sisco, Daphne T. Adelman, William H. Ludlam, Asi Haviv, Dana Gelbaum, Shuqian Liu, Susan D. Mathias, Lizheng Shi

**Affiliations:** 1 Memorial Sloan Kettering Cancer Center, 1275 York Ave, Box 419, New York, NY 10065 USA; 2 Acromegaly Community, Grove, OK USA; 3 Northwestern University, Chicago, IL USA; 4 Chiasma, Inc., Waltham, MA USA; 5 Tulane University, New Orleans, LA USA; 6 Health Outcomes Solutions, Winter Park, FL USA

**Keywords:** Acromegaly, Somatostatin receptor ligands, Treatment satisfaction, Patient reported outcomes, Questionnaire, Concordance

## Abstract

**Background:**

Acromegaly patients, even those with IGF-1 values within the normal range receiving somatostatin receptor ligands (SRLs), often suffer from significant symptoms. It is not known to what extent patients’ medical providers are aware of the frequency and severity of acromegaly symptoms or level of treatment satisfaction with SRLs. This study sought to examine the concordance between outcomes reported by acromegaly patients treated with long-acting SRLs and those perceived by their medical provider.

**Methods:**

US acromegaly patients on a stable dose of SRL and seen by their medical provider in the past year completed an online survey which included the Acro-TSQ. Their medical providers were interviewed about the perception of their patient’s symptoms, level of control, and general health, and completed relevant portions of the Acro-TSQ. Concordance between patient and medical provider reported data was examined.

**Results:**

Medical providers reported that their patients experienced acromegaly symptoms on a regular basis, however, there was poor agreement between patients and medical providers on the frequency, severity, and pattern of symptoms, as well as on the severity of injection site reactions and multiple domains of the Acro-TSQ, with patients generally reporting symptoms and injection site reactions more often and with higher severity than medical providers.

**Conclusions:**

Medical providers were aware that their patients who were receiving a stable dose of SRL regularly experienced acromegaly symptoms. Addressing discordance in patient- and medical provider-reported frequency and severity of acromegaly symptoms and injection site reactions by facilitating better communication may improve care of acromegaly patients.

## Introduction

Acromegaly is an endocrine disorder characterized by excess production of growth hormone (GH) and insulin-like growth factor (IGF-1); it can result in changes in facial appearance and enlargement of the hands and feet, among other signs and symptoms [1–4]. In addition, acromegaly patients may experience significant economic burden and marked detriments to health-related quality of life from both the disease and its treatment [5–9], including potential side effects associated with somatostatin receptor ligands (SRLs) [10, 11], which are the most common first-line medical therapy [12, 13]. Even when medical therapy successfully regulates production of GH and IGF-1 to within thresholds of normal, acromegaly symptoms can interfere with daily life, leisure, and work [8].

Effective communication between medical providers and acromegaly patients can enhance the patient experience and promote treatment adherence [14, 15]. Multiple studies have shown that in patients with chronic conditions, good communication with their provider is associated with better outcomes and lower health expenditures [16–18]. However, communication in acromegaly care can be less than optimal [19]. There may be several reasons for discordance between acromegaly patients and providers, including insufficient time during the visit to fully discuss symptoms and treatment, or patient reluctance to ask questions or share details about the impact of acromegaly on their lives with their medical provider [20].

Discordance between patient-provider assessments has been described in other diseases. In a 2001 study in Finland of patients who visited a general practitioner (GP) because of pain, a comparison of pain intensity ratings (measured by the Visual Analogue Scale [VAS]) between patients and their GPs showed that GPs tended to under-estimate the intensity of patients’ pain. The disagreement was higher at the highest levels of patient-reported pain severity: at the highest grade of pain intensity as perceived by the patients, GPs rated the pain lower than did their patient in 82% of the instances [21]. In rheumatoid arthritis (RA), discordance between patient versus medical provider assessments of disease activity occurs frequently. A 2017 study asked 350 patients and their medical providers to complete a global assessment of disease activity (using VAS); authors observed discordance in 33% of patients, and in all but 11 cases patients rated their disease activity higher than did their medical providers (‘negative’ discordance) [22]. A meta-analysis of 12 studies calculated that the average discordance in patient-medical provider global assessment of RA was 43% (range 25–76%) [23].

Concordance between patient-provider perceptions of symptoms and aspects of treatment (including injection-site reactions and side effects) of acromegaly has not previously been examined. Understanding whether discordance exists and, if so, in what areas or to what extent would assist in addressing related unmet needs for effective acromegaly treatment. This study sought to evaluate the concordance between outcomes reported by US acromegaly patients treated with long-acting SRLs and those perceived by their treating endocrinology medical provider.

## Methods

### Study type and patient population

This cross-sectional, US-based study involved acromegaly patients recruited by Acromegaly Community, Inc. (http://www.acromegalycommunity.org) using an Institutional Review Board (IRB)-approved invitation letter and advertisement posted on its Facebook page and by clinical practices in the US. Adult patients (aged 18 to < 95 years) who self-reported an acromegaly diagnosis which was subsequently confirmed by a knowledge screening questionnaire based on current medications and doses were eligible. In addition, patients were required to be currently receiving injectable SRLs, Sandostatin^®^ LAR (octreotide) or Somatuline^®^ Depot (lanreotide), for ≥ 12 months with no change in dose at the time of or since their last office visit, to have seen their treating acromegaly medical provider within the past 12 months (± 2 months), to read and understand English, to live and receive acromegaly treatment in the US, and be willing to provide signed informed consent. Patients were excluded if they were a previous or current participant in a Mycapssa^®^ (octreotide capsules) trial or used Pegvisomant (Somavert^®^) monotherapy or Pasireotide (Signifor^®^). This study was approved by the IRB at Tulane University.

Patients completed an online survey focusing on disease characteristics and management (including their perception of biochemical control), the occurrence, control, and severity of symptoms, adverse reactions, and general health. Patients also completed the Acro-TSQ (an acromegaly-specific PRO assessing symptoms and GI interference, treatment satisfaction, injection site interference, emotional reaction, and treatment convenience) [24]. Finally, patients were asked to provide contact information for their designated acromegaly medical provider(s).

Medical providers were contacted and asked to participate in a telephone interview about the patient who provided their contact information and were asked to have the patient’s medical chart available during the interview. Medical providers were asked about their own practice, including the number of years they had practiced, how long they had treated acromegaly patients, and the type of healthcare facility in which they practiced. The gender and specialty of each medical provider were also recorded. The interview inquired about the medical providers’ perception of the specific patient’s acromegaly and treatment, including: latest IGF-1 test results (if available), acromegaly signs and symptoms (including severity and pattern of symptoms), injection related signs, symptoms, reactions, the level of acromegaly control, the patient’s satisfaction with treatment, and a general health rating. Medical providers also completed four of the six domains of the Acro-TSQ that investigators thought could be reliably answered.

### Statistical analysis

Analyses of acromegaly patient data included descriptive analyses (frequencies and percentages or means, standard deviations [SDs], and ranges) on demographic (age, gender) and clinical characteristics (duration of disease, current treatment, history of other therapies), routine management, disease activities, adverse drug reactions, treatment satisfaction, and general health rating, as well as descriptive analyses on Acro-TSQ domain scores (Symptom interference, GI Interference, Treatment Satisfaction, Injection Site Interference, Emotional Reaction, and Treatment Convenience). Domain scores range from 0 (most symptomatic/interference) to 100 (least symptomatic/interference).

Analyses of medical provider-reported data included descriptive analyses (frequencies and percentages or means, SD, and ranges) on demographic (age, gender) and professional characteristics (medical specialty, years in practice, clinic setting), and reported disease activity, adverse drug reactions, treatment satisfaction, and general health rating, and descriptive analyses on Acro-TSQ domain scores (except for the domains of Emotional Reaction and Treatment Convenience, which were not assessed by medical providers).

To examine concordance between patient- and medical provider-reported data, cross-tabulation tables were created to compare responses. To assess the level of agreement in discrete data, Cohen’s kappa (for nominal data) and weighted kappa (for ordinal) were used. Criteria suggested by Landis and Koch [25] were used to characterize the level of agreement reflected by observed kappa values. For continuous data, Lin’s [26] concordance correlation coefficients (CCCs) and two-way mixed effects consistency intraclass correlation coefficients (ICCs) [27] were used; Lin’s criteria was used to characterize how these metrics reflect the strength of the concordance and reliability between responses [27].

## Results

### Demographic and clinical characteristics of patients and medical providers

Of 146 eligible patients identified, a total of 112 (77%) signed consent forms and provided contact information for their medical providers, and 105 (94%) patients completed the survey. Among the medical providers contacted, 49 responded (44%) and 47 of those 49 (96%) completed interviews. Therefore, data from 47 patient-medical provider pairs were available for inclusion in the analysis.

As shown in Table 1, 83% of patients were female, and the mean (SD) age was 49 (12.3) years. The mean (SD) duration of acromegaly was 10 (8.1) years; 47% were using octreotide and 53% lanreotide, and most were receiving low to middle doses. SRL monotherapy was common (62%); 45 of 47 had pituitary surgery (9 also had radiotherapy, 36 did not). On average, patients reported having about three medical provider visits for acromegaly in the past year. The mean (SD) medical provider-reported IGF-1 was 0.85 (0.56) ULN, with 79% having an IGF-1 ≤ 1 ULN.

**Table 1:** Demographic and clinical characteristics

Characteristic	Results
Patients, N = 47	
Female, % (N)	83 (39)
Age, mean ± SD (years)	49 ± 12.3
Duration of acromegaly, mean ± SD (years)	10 ± 8.1
Current SRL, % (N)	
Octreotide	47 (22)
Low dose (< 20 mg total/month)	32 (7)
Middle dose (20 mg to < 30 mg total/month)	23 (5)
High dose (≥ 30 mg total/month)	46 (10)
Lanreotide	53 (25)
Low dose (< 90 mg total/month)	36 (9)
Middle dose 90 mg to < 120 mg total/month)	32 (8)
High dose (≥ 120 mg total/month)	32 (8)
Medications for acromegaly, % (N)	
SRL Only	62 (29)
SRL + pegvisomant (Somavert®)	17 (8)
SRL + cabergoline (Dostinex® or Cabaser®)	13 (6)
SRL + GH-receptor + dopamine	4 (2)
Unknown	4 (2)
Procedure, % (N)	
Pituitary surgery only	77 (36)
Radiotherapy only	0 (0)
Both pituitary surgery and radiotherapy	19 (9)
Neither pituitary surgery or radiotherapy	4 (2)
Time since pituitary surgery, mean ± SD (years)	9 ± 8.2
Number of medical provider visits in past year, mean ± SD	3 ± 2.1
IGF-1, mean ± SD (ULN)	0.85 ± 0.56
IGF-1 < = 1 ULN, % (N)	79 (37)
IGF-1 > 1 ULN, % (N)	21 (10)
Medical providers, N = 47	
Female, % (N)	60 (28)
Years in practice, mean ± SD	20 ± 12.7
Clinic setting	
Academic hospital	62 (29)
Community hospital/private	38 (18)

*GH *growth hormone, *IGF-1* insulin-like growth factor 1, *SD* standard deviation, *SRL* somatostatin receptor ligands, *ULN* upper limit of normal

Among the 47 medical providers included, 60% were female. As a group, the mean (SD) years in practice was 20 (12.7); 62% practiced in an academic setting with the remaining 38% in a community hospital or private practice.

### Patient-reported results

The most common symptoms reported by patients were acro-fog (general forgetfulness or short-term memory loss) and joint pain (81% for both), soft tissue swelling (79%), fatigue/weakness/tiredness (77%), and headache (72%) (Table 2). Symptoms most often reported as being severe included joint pain (34% of those who reported joint pain rated it as severe), headache (29% severe), fatigue/weakness/tired (28% severe), and acro-fog (26% severe) (Table 2).

**Table 2:** Frequency and severity of symptoms reported by medical providers and patients (N = 47)

Symptom	Reported by patient				Reported by medical providers					
	Experienced symptom yes % (N)	Mild %^a^ (n)	Moderate %^a^ (n)	Severe %^a^ (n)	Experienced symptom yes % (N)	Mild %^a^ (n)	Moderate %^a^ (n)	Severe %^a^ (n)	No severity %^a^ (n)	Not sure %^a^ (n)
Headache^b^	72 (34)	50 (17)	21% (7)	29 (10)	62 (29)	28 (8)	52 (15)	14 (4)	0 (0)	7 (2)
Fatigue/ weakness/ feeling tired^c^	77 (36)	22 (8)	50 (18)	28 (10)	92 (43)	28 (12)	51 (22)	16 (7)	0 (0)	5 (2)
Excess sweating^c^	64 (30)	53 (16)	30 (9)	17 (5)	40 (19)	42 (8)	32 (6)	5 (1)	0 (0)	21 (4)
Joint pain^b^	81 (38)	29 (11)	37 (14)	34 (13)	75 (35)	29 (10)	40 (14)	20 (7)	6 (2)	6 (2)
Swelling of soft tissue^b^	79 (37)	38 (14)	38 (14)	24 (9)	51 (24)	42 (10)	42 (10)	13 (3)	4 (1)	0 (0)
Carpal tunnel syndrome^b^	66 (31)	65 (20)	29 (9)	7 (2)	21 (10)	20 (2)	10 (1)	10 (1)	0 (0)	60 (6)
Vision problem^b^	53 (25)	56 (14)	24 (6)	20 (5)	11 (5)	40 (2)	0 (0)	0 (0)	20 (1)	40 (2)
Snore^b^	60 (28)	57 (16)	25 (7)	18 (5)	49 (23)	30 (7)	26 (6)	9 (2)	0 (0)	35 (8)
Acro-Fog^c^	81 (38)	29 (11)	45 (17)	26 (10)	51 (24)	33 (8)	29 (7)	13 (3)	4 (1)	21 (5)

^a^Percent is out of total N reported as experiencing symptom

^b^Weighted kappa < 0 (poor)

^c^Weighted kappa 0 to 0.20 (slight)

Symptoms were most commonly reported as either occurring constantly or towards the end of the cycle. Acro-fog was most commonly reported as occurring constantly (87% of patients who reported acro-fog reported it occurring constantly), followed by fatigue, weakness, or tiredness (81%), and snoring (75%, Table 3). Symptoms most commonly reported by patients as occurring or worsening near the end of the cycle were headache (29%), joint pain (29%), swelling of the soft tissue (27%), and excess sweating (23%, Table 3). Some symptoms were rarely reported by patients as occurring or worsening at the end of the cycle, including vision problems (4%), snoring (7%), and acro-fog (8%). When asked about their perception of symptom control, 38% of patients indicated that their symptoms were “well controlled”, while 43% reported that they were “partially controlled” and 17% reported that they were “not controlled” (data not shown).

**Table 3:** Pattern of symptoms reported by medical providers and patients (N = 47)

Symptom	Reported by patients						Reported by medical providers					
	Experienced symptom yes % (N)	Constant %^a^ (n)	Right after injection %^a^ (n)	Middle of cycle %^a^ (n)	End of cycle %^a^ (n)	Not sure %^a^ (n)	Experienced symptom yes % (N)	Constant %^a^ (n)	Right after injection %^a^ (n)	Middle of cycle %^a^ (n)	End of cycle %^a^ (n)	Not sure %^a^ (n)
Headache^b^	72.3 (34)	50 (17)	6 (2)	0 (0)	29 (10)	15 (5)	62 (29)	52 (15)	0 (0)	0 (0)	10 (3)	38 (11)
Fatigue/ weakness/ feeling tired^c^	76.6 (36)	81 (29)	0 (0)	6 (2)	8 (3)	6 (2)	92 (43)	56 (24)	2 (1)	0 (0)	9 (4)	33 (14)
Excess sweating^c^	63.8 (30)	47 (14)	7 (2)	3 (1)	23 (7)	20 (6)	40 (19)	42 (8)	0 (0)	0 (0)	16 (3)	42 (8)
Joint pain^c^	80.9 (38)	63 (24)	0 (0)	0 (0)	29 (11)	8 (3)	75 (35)	57 (20)	0 (0)	0 (0)	9 (3)	34 (12)
Swelling of soft tissue^c^	78.7 (37)	51 (19)	5 (2)	3 (1)	27 (10)	14 (5)	51 (24)	58 (14)	0 (0)	0 (0)	4 (1)	38 (9)
Carpal tunnel syndrome^b^	66.0 (31)	65 (20)	3 (1)	0 (0)	16 (5)	16 (5)	21 (10)	10 (1)	0 (0)	0 (0)	0 (0)	90 (9)
Vision problem^c^	53.2 (25)	64 (16)	0 (0)	4 (1)	4 (1)	28 (7)	11 (5)	20 (2)	0 (0)	0 (0)	0 (0)	80 (4)
Snore^c^	59.6 (28)	75 (21)	4 (1)	0 (0)	7 (2)	14 (4)	49 (23)	52 (12)	0 (0)	0 (0)	0 (0)	48 (1)
Acro-Fog^c,d^	80.9 (38)	87 (33)	0 (0)	0 (0)	8 (3)	5 (2)	50 (23)	39 (9)	0 (0)	0 (0)	4 (1)	57 (13)

^a^Percent is out of total N reported as experiencing symptom

^b^Weighted kappa 0 to 0.20 (slight)

^c^Weighted kappa < 0 (poor)

^d^Total N = 46 for Medical provider

Patients reported several different types of injection site reactions, including pain during the injection (90%) or several hours (72%) or days (53%) after, as well as nodules (68%), swelling (51%), bruising (47%) and scar tissue or hardness of the skin (47%, Table 4). These reactions were most commonly rated as either mild (range 27–56%) or moderate (range 27–56%).

**Table 4:** Frequency and severity of injection site reactions reported by medical providers and patients (N = 47)

Reactions^a^	Reported by patients				Reported by medical providers				
	Experienced reaction yes % (N)	Mild %^b^ (n)	Moderate %^b^ (n)	Severe %^b^ (n)	Experienced reaction yes % (N)	Mild %^b^ (n)	Moderate %^b^ (n)	Severe %^b^ (n)	Not sure %^b^ (n)
Pain during injection^c^	90 (42)	52 (22)	31 (13)	17 (7)	66 (31)	36 (11)	10 (3)	3 (1)	52 (16)
Pain several hours after injection^d^	72 (34)	53 (18)	38 (13)	9 (3)	55 (26)	23 (6)	8 (2)	0 (0)	69 (18)
Pain several days after injection^d^	53 (25)	56 (14)	28 (7)	16 (4)	45 (21)	14 (3)	0 (0)	0 (0)	86(18)
Bruising^c^	47 (22)	73 (16)	27 (6)	0 (0)	43 (20)	35 (7)	5 (1)	0 (0)	60 (12)
Swelling^d^	51 (24)	50 (12)	46 (11)	4 (1)	36 (17)	24 (4)	0 (0)	0 (0)	77 (13)
Nodules^d^	68 (32)	31 (10)	56 (18)	13 (4)	43 (20)	20 (4)	5 (1)	0 (0)	75 (15)
Scar tissue/ hardness of the skin^c^	47 (22)	27 (6)	55 (12)	18 (4)	34 (16)	25 (4)	0 (0)	0 (0)	75 (12)

^a^At the injection site

^b^Percent is out of total N reported as experiencing reaction

^c^Weighted kappa 0 to 0.20 (slight)

^d^Weighted kappa < 0 (poor)

**Fig. 1:**
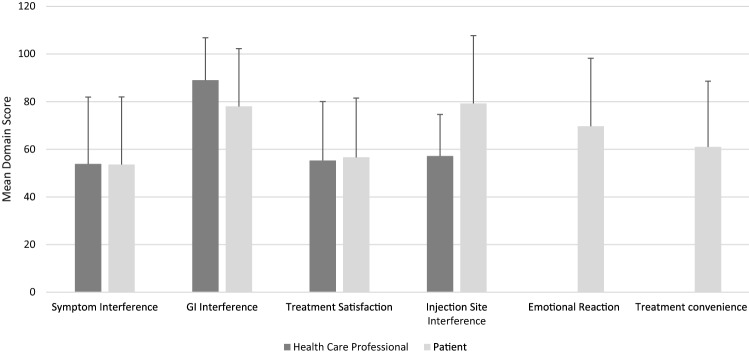
Acro-TSQ domain scores (N = 47). Due to “not sure” responses, sample sizes for medical provider-based domain scores are lower for Symptom Interference (N = 41) and Injection Site Interference (N = 7). Domain scores can range from 0 (most symptomatic/interference) to 100 (least symptomatic/interference)

Mean (SD) Acro-TSQ domain scores appear in Fig. 1 and range from 54 (28) for Symptom Interference to 79 (28) for Injection Site Interference. The mean (SD) rating for self-reported general health rating was 65 (19).

### Medical provider-reported results

Medical providers most frequently reported fatigue/weakness/tired (92%) in their patients, followed by joint pain (75%), and headache (62%); the percent of patient symptoms medical providers rated as severe ranged from 0% (vision problem) to 20% (joint pain) (Table 2).

Medical providers commonly reported that they were not sure when during the cycle their patients’ symptoms occurred (range 33–90%) (Table 3). When asked about the level of disease control, 28% of medical providers indicated that their patient’s symptoms were well-controlled, 64% said they were “partially controlled” and 6% said they were “not controlled” (data not shown).

Medical providers reported each type of injection-site reaction less often than did patients, with the largest discrepancies in nodules (43% by medical providers vs 68% by patients) and pain during the injection (66% vs 90% by patients) (Table 4). It was common for medical providers to report that they were unsure of the severity of their patients’ symptoms (range 52% for pain during injection to 86% for pain several days after).

Of the 4 Acro-TSQ domain scores completed by medical providers, two (GI Interference and Injection Site Interference) had reduced sample sizes (N = 41 and 7, respectively) due to medical providers responding “not sure” to items included in those domains. Mean (SD) domain scores ranged from 54 (28) for Symptom Interference to 89 (18) for GI Interference (Fig. 1). The mean (SD) general health rating of patients as rated by medical providers was 68 (18).

### Concordance between patient-reported and medical provider-reported results

Concordance was generally low in the occurrence, severity, and pattern of symptoms, as represented by low and (frequently) not statistically significant weighted kappa statistics (Tables 2, 3). As reflected by weighted kappa statistics in absolute value ≤ 0.007 (Table 4), concordance was very low in the reporting of the frequency and severity of Injection Site Reactions. ICCs on the domain scores between patients and medical providers reflected poor (< 0.5) to moderate (0.5 to 0.75) reliability: 0.20 (95% confidence interval [CI] 0.04, 0.60) for GI interference, 0.57 (0.37, 0.74) for Treatment Satisfaction, 0.61 (0.43, 0.77) for Symptom Interference, and 0.17 (0.0, 0.96 for Injection Site Reaction (although it was based on only 7 patient-medical provider pairs). All CCCs indicated poor concordance. Medical providers rated their patients’ general health slightly higher than patients did, on average (68 vs 65). The ICC (95% CI) for general health scores was 0.12 (0.01, 0.66).

## Discussion

This study examined the level of concordance between patient- and medical provider-reported outcomes for acromegaly patients on a stable dose of SRL. Patients reported experiencing a variety of symptoms that were often moderate to severe that most often occurred constantly; multiple symptoms were reported to occur or worsen at the end of the cycle. Patients also reported several different types of injection site reactions. Medical providers, too, reported that their patients experience a variety of symptoms and injection site reactions.

However, findings from the patient- and medical provider-reported data reveal discordance in the frequency and severity of symptoms and injection site reactions. Medical providers were less likely than patients to report headache, excess sweating, joint pain, swelling, carpal tunnel syndrome, vision problems, snoring, and acro-fog, and more often reported fatigue/weakness/tired. Additionally, medical providers rated symptoms as “severe” less often than patients for all symptoms except carpal tunnel syndrome. Medical providers also frequently reported that they were “not sure” when patients experienced these symptoms. Although the difference in the relative frequency of symptoms and their severity reported by patients and medical providers were not tested for statistical significance, the low kappa statistics reflect the generally poor agreement between medical providers’ and patients’ responses of symptom frequency, severity, and pattern. Regarding treatment, medical providers reported fewer injection site reactions than patients, and in many cases, were unaware of the severity of the reactions.

Under current guidelines, for patients receiving SRLs whose IGF-1 ≤ 1 ULN, adjustments to treatment are not considered necessary. Further, it is not known whether SRL dose titration in patients whose IGF-1 ≤ ULN can reduce symptom burden. However, our data show that these patients are still symptomatic and that the frequency and severity of symptoms is often unrecognized by their treating physician. This suggests an unmet need.

The discordance in patient- and medical provider-reported outcomes observed in other conditions has been shown to be associated with other aspects of care and patient outcomes. In RA, patients who rate their disease activity higher than their medical providers (‘negative’ discordance) may also report higher levels of work impairment and achieve remission less frequently that those whose responses are concordant with those of their medical providers [28, 29]. This may reflect poor communication or simply a difference in how patients and medical providers perceive how RA symptoms affect patients’ lives. Improvements in disease control can reduce this discordance, but may not eliminate it; the presence of symptoms, like pain, in patients whose disease is considered to be in control may cause them to continue to consider their disease activity to be more severe than would their medical provider [28]. Discordance in pain perceptions between patients and medical providers has been shown to be more common among patients with worse pain and poorer physical functioning [30]. Although it is not clear how concordance may affect subsequent health status, good patient-provider communication can promote greater treatment adherence to pain therapies [31].

Some discordance regarding symptom and treatment burden between medical provider and acromegaly patients may be reduced through better communication. Regular discussion of SRL treatment may contribute to better treatment experiences and help address aspects which can make adherence challenging [14]. It is possible that those who do discuss their experiences during monthly injections with a nurse may not do so when seeing their treating physician. Previous research has demonstrated that within acromegaly, the quality of the patient-medical provider relationship can influence how willing patients are to discuss aspects of their disease and treatment [20].

This is the first study to our knowledge to examine concordance between acromegaly patients and their medical providers regarding disease burden, treatment satisfaction, and general health. However, these results should be viewed in light of several limitations. First, patients were recruited by social media, were receiving a stable dose of injectable SRL, and had seen their treating physician within the past year. It is unclear to what extent these findings are generalizable to other patient populations with acromegaly. Second, there may be some recall bias (when patients do not remember previous experiences accurately or omit details), as all data were based on self-report. Medical providers relied on chart notes and recollection from prior visits. Lastly, with a 44% response rate, there is potential for some non-response bias.

The results of this study reveal that medical providers recognize that their patients experience acromegaly symptoms—frequently with a severity described as moderate or severe—but may keep them on a stable dose of SRL, even though the majority are receiving low to medium doses (< 30 mg total/month for octreotide and < 120 mg total/month for lanreotide). Additionally, compared with patients, medical providers tended to report fewer acromegaly symptoms and injection site reactions, and rated general health higher.

The data presented in our study provide a platform for the acromegaly patients’ experience living with this chronic condition, hopefully raising awareness about the discrepancies between the medical providers’ and patients’ assessment of disease outcomes. Our findings show that management of patients with acromegaly should incorporate regular open communication between doctor and patient, and highlights the need to incorporate patient reported outcomes in the management of acromegaly. Treatments should aim to focus on symptomatic as well as biochemical control. Improved communication between patients and medical providers as well as therapies that more completely control symptoms could improve acromegaly care.
